# Stretchable Surface Electrode Arrays Using an Alginate/PEDOT:PSS-Based Conductive Hydrogel for Conformal Brain Interfacing

**DOI:** 10.3390/polym15010084

**Published:** 2022-12-25

**Authors:** Sungjun Lee, Kyuha Park, Jeungeun Kum, Soojung An, Ki Jun Yu, Hyungmin Kim, Mikyung Shin, Donghee Son

**Affiliations:** 1 School of Electrical and Electronic Engineering, Yonsei University, Seoul 03722, Republic of Korea; 2 Center for Bionics of Biomedical Research Institute, Korea Institute of Science and Technology, Seoul 02792, Republic of Korea; 3 Department of Electrical and Computer Engineering, Sungkyunkwan University (SKKU), Suwon 16419, Republic of Korea; 4 Division of Bio-Medical Science & Technology, KIST School, University of Science and Technology, Seoul 02792, Republic of Korea; 5 Department of Intelligent Precision Healthcare Convergence, Sungkyunkwan University (SKKU), Suwon 16419, Republic of Korea; 6 Department of Biomedical Engineering, Sungkyunkwan University (SKKU), Suwon 16419, Republic of Korea; 7 Department of Superintelligence Engineering, Sungkyunkwan University (SKKU), Suwon 16419, Republic of Korea

**Keywords:** stretchable electronics, soft electronics, implantable electronics, brain interface, electrocorticogram, conductive hydrogel

## Abstract

An electrocorticogram (ECoG) is the electrical activity obtainable from the cerebral cortex and an informative source with considerable potential for future advanced applications in various brain-interfacing technologies. Considerable effort has been devoted to developing biocompatible, conformal, soft, and conductive interfacial materials for bridging devices and brain tissue; however, the implementation of brain-adaptive materials with optimized electrical and mechanical characteristics remains challenging. Herein, we present surface electrode arrays using the soft tough ionic conductive hydrogel (STICH). The newly proposed STICH features brain-adaptive softness with Young’s modulus of ~9.46 kPa, which is sufficient to form a conformal interface with the cortex. Additionally, the STICH has high toughness of ~36.85 kJ/mm^3^, highlighting its robustness for maintaining the solid structure during interfacing with wet brain tissue. The stretchable metal electrodes with a wavy pattern printed on the elastomer were coated with the STICH as an interfacial layer, resulting in an improvement of the impedance from 60 kΩ to 10 kΩ at 1 kHz after coating. Acute in vivo experiments for ECoG monitoring were performed in anesthetized rodents, thereby successfully realizing conformal interfacing to the animal’s cortex and the sensitive recording of electrical activity using the STICH-coated electrodes, which exhibited a higher visual-evoked potential (VEP) amplitude than that of the control device.

## 1. Introduction

Brain interface technology is a bio-electronic bridging platform for monitoring brain activity [1,2,3,4,5] or modulating brain function [6,7,8,9,10,11,12,13,14,15,16,17] by connecting electronic devices to the neurological system. Owing to its technological-convergence-related benefits [18,19,20,21,22,23,24,25], brain interface platforms could be applied to various advanced biomechatronic-associated areas that include: (i) biomedical applications, such as the diagnosis and therapy of neuropathy, daily biomonitoring, healthcare, recovery of brain function from trauma or injury, rehabilitation, and prosthetics for quadriplegia and motor or sensory dysfunction; (ii) human–machine connection for the remote control of objects and avatar robotics; (iii) human enhancement technology, such as memory reinforcement and cognitive function extension; and (iv) tangible user-experience media, such as immersive virtual or augmented reality, metaverse contents, and realistic interactive gaming.

Recently, the expected ripple effect and utilization of brain interface technology have become significant in the biomedical engineering and neuroscience domains. In particular, the acquisition of the electrical activity generated from cranial neurons using large-area, high-density integrated multielectrode arrays permits the digitization of diagnostic information, such as the location, range, and degree of brain damage, qualitatively and quantitatively, by capturing the pathological neurosignals caused by brain disorders [26,27,28,29,30,31,32,33,34,35,36,37]. Furthermore, the real-time recording of neural activity progression enables detailed observations of the developmental pattern of pathological symptoms and the therapeutic effects of neurostimulation [8,38]. Ultimately, the multidimensional spatiotemporal mapping of the genesis, propagation, and distribution of electrical activity from the brain with high resolution and fidelity facilitates advanced neuroscience research by identifying the brainwave patterns that involve and control physical activities, such as thinking, association, sensation, perception, cognition, behavior, motion, exercise, learning, memory, and noegenesis [27,28,29,30,31,39,40,41,42,43,44,45,46,47,48,49,50,51,52,53,54,55,56].

To pursue the aforementioned objectives, sensor systems for acquiring brain-activity-related electrophysiological information were developed and actively researched. These brain-interfacing sensor systems acquire the electrical brain activity propagated from cranial nerves through the following approaches based on the access to brain nerves: (i) electroencephalography (EEG), (ii) electrocorticography (ECoG), (iii) local field potential (LFP) analysis, and (iv) single-neuron spiking [1]. In particular, the ECoG monitoring platform has various advantages compared to the others. Because electrical signals are recorded from intracranial-implanted electrodes in this approach, an intimate bio-electronic interface with excellent impedance properties could be readily formed via close contact with neurons, leading to the acquisition of brain activity signals with a higher signal-to-noise ratio (SNR) than that of EEG platforms that are often disturbed by hair [57]. In terms of diagnosing brain disease in clinical settings, ECoG directly provides more diagnostically significant information than that derived from EEG methods such as high-frequency oscillation (HFO) as a presymptom of epileptic seizure frequently not transmitted across the skull and cannot be detected from the scalp [27,28,29,31,33]. Compared to the probe-type electrodes penetrating brain tissue with high modulus and stiffness for recording LFPs or spikes [4,5,58,59,60], ECoG devices are safe and biocompatible tools that could be used for prolonged durations owing to their non-invasiveness into the brain tissue, thereby advantageous for large-area brain monitoring with multielectrode arrays in a matrix formation. Implementing the spatiotemporal topography of ECoG activity with high resolution, fidelity, and an SNR with large-area coverage is critical for the detailed documentation and imaging of brain activity development, tracing segmentalized functional brain maps for use in various brain-associated applications, and in-depth neuroscience studies [27,28,29,30,31]. Advanced surface electrode devices have been developed for ECoG technology using inorganic materials, such as metals and ceramics, to conveniently achieve high scalability for ultrafine patterning and high integration density, large-area uniformity, high yield, high throughput, mass productivity, guaranteed electrical performance, and long-term durability [27,28,29,30,31,61]. Ultrathin-film devices that are sub-tens of micrometers in thickness exhibit mechanical deformation characteristics, such as flexibility, bendability, and foldability, by drastically lowering the stiffness and bending the radius of the system, and their mesh-like electronic pattern improves adaptability by lowering the effective surface moduli [62]. The wavy interconnect pattern of electronic devices imparts mechanical stretchability to the systems, and stretchable thin-film devices printed on the soft stretchable substrates could be integrated with biological tissue as artificial electronic integuments. 

However, these rigid inorganic materials and polymers with high Young’s moduli (10^2^ MPa~10^2^ GPa) have intrinsic limitations in achieving adaptive conformal interfacing with the cortex, which is the softest tissue (almost 1 kPa Young’s modulus) in the human body [63]. In addition, connecting a biometric system operated with ion transportation with electrical sensing devices without ionic conductivity also has fundamental limitations to implementing an adaptive bio-electronics interface with low electrochemical impedance [63,64]. There have been massive efforts to introduce soft conductive interfacial organic materials to be used in the form of a coating layer on the electrode surface to bridge electronic devices and biological tissues while matching surface moduli for adaptive conformal bio-electronic interfaces [65,66,67,68,69,70,71,72,73,74,75,76,77,78,79,80]. However, the implementation of functional brain-interfacing materials capable of both high toughness and low Young’s moduli, which desirably feature high compatibility to soft brain tissue and high durability while being coated on an elastic electrode surface, is still challenging.

**Figure 1:**
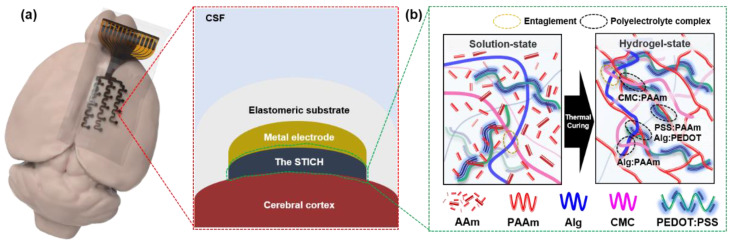
Illustrations of the stretchable surface electrode arrays using the soft tough ionic conductive hydrogel (STICH) for adaptive conformal brain interfacing. (**a**) In vivo demonstration of ECoG monitoring using an animal model, in which the electrode device is mounted on the cerebral cortex of a rat (left). The STICH layer coated on the metal electrode channel provides adaptive brain-electronics interfacing owing to its soft modulus and conformal contact with the cortex (right). (**b**) Three types of gel networks: intertwined, joined via covalent bonding (black ovals), and showing entanglements (yellow ovals) between the amine groups on polyacrylamide chains and the carboxyl groups on the Alg and CMC chains. (acrylamide, AAm; alginate, Alg; carboxymethyl cellulose, CMC; polyacrylamide, PAAm; poly(3,4-ethylenedioxythiophene):poly(styrenesulfonate), PEDOT:PSS).

Herein, we developed stretchable surface electrode arrays using the newly proposed soft tough ionic conductive hydrogel (STICH) composites with a triple-network structure consisting of poly (3,4-ethylenedioxythiophene):polystyrene sulfonate (PEDOT:PSS), alginate (Alg), polyacrylamide (PAAm), and carboxymethyl cellulose (CMC) for adaptive conformal brain interfacing (Figure 1). Alg, CMC, and PAAm are most widely used for preparing hydrogel-based applications which feature low moduli, high biocompatibility, good processability, and good ionic conductivity via polyelectrolyte complexes (PECs) between polyanions and polycations [81,82]. In addition, compared to other hydrogels, Alg-based hydrogels have less dynamic change properties due to heat, so when used as implant electrodes later, significant changes in the adjusted mechanical properties are not expected [83,84]. We fabricated and optimized the STICH for brain interfacing using these biomaterials and employed the new composite material for ECoG monitoring. The in vivo ECoG monitoring capability of our stretchable electrodes with the STICH was illustrated (Figure 1a). The thermo-curable STICH comprises a densely networked structure featuring ionic interactions and entanglements (Figure 1b).

## 2. Materials and Methods

### 2.1. Preparation of the Alg, CMC, and PAAm-Based Conductive Hydrogels

The hydrogel materials were prepared using our previously reported one-pot synthesis procedure for Alg/CMC/PAAm-based conductive hydrogels [83]. Initially, polyanion precursors in various proportions (100% Alg (sodium alginate, Sigma-Aldrich, Inc., St. Louis, MO, USA), 100% carboxymethylcellulose sodium salt (CMC, low viscosity, Sigma-Aldrich, Inc., St. Louis, MO, USA), and a 1:1 Alg:CMC mixture were mixed with deionized water at 2 wt.%. Subsequently, acrylamide (AAm, suitable for electrophoresis, ≥99%, Sigma-Aldrich, Inc., St. Louis, MO, USA) (16 wt.% of the polyanion precursor) was dissolved in the different polyanion solutions, which were then vigorously stirred for 30 min until homogeneous solutions were obtained. *N,N′*-methylenebisacrylamide (MBAA, powder, for molecular biology, suitable for electrophoresis, ≥99.5%, Sigma-Aldrich, Inc., St. Louis, MO, USA) (10^−2^ wt.% of the total deionized water content), Clevios™ PH 1000 (aqueous PEDOT/PSS dispersion, blue liquid, Heraeus, Ohio, Dayton, USA) (20 wt.% of the total deionized water content), and ammonium persulfate (APS, ACS reagent, ≥98.0%, Sigma-Aldrich, Inc., St. Louis, MO, USA) (6 wt.% of the total AAm content) were then added to the mixed solutions, followed by *N,N,N′,N′*-Tetramethylethylenediamine (TEMED, BioReagent, suitable for electrophoresis, ~99%, Sigma-Aldrich, Inc., St. Louis, MO, USA) (0.1 wt.% of the deionized water). The resulting solutions were transferred to manually manufactured molds and maintained at 60 °C for 30 min. The compositions of the prepared STICH and control materials are listed in Table 1.

### 2.2. Physicochemical Characterization of the Alg, CMC, and PAAm-Based Conductive Hydrogels

The morphology of the STICH and control materials were analyzed with scanning electron microscopy (SEM; JSM-7600F Schottky-field-emission scanning electron microscope; JEOL USA, Inc., Peabody, MA, USA). To investigate the chemical compositions of the Alg, CMC, and PAAm in the STICH as well as their interactions, the STICH and the controls were analyzed with attenuated-total-reflectance Fourier transform infrared (ATR-FTIR) spectroscopy (Bruker IFS-66/S, TENSOR27, Bruker, Republic of Korea).

### 2.3. Mechanical and Rheological Characterization of the Alg, CMC, and PAAm-Based Conductive Hydrogels

To measure the toughness and Young’s moduli of the STICH and controls, cylindrical samples were fabricated using a premade cylindrical mold (2.5 × 2.5 × 30 mm^3^). The measurements were performed using a universal testing machine (UTM; 34SC-1 Single Column Table Model, INSTRON, Norwood, MA, USA) in two modes: continuous strain at a rate of 20 mm/min and cyclic stretching (approximately 10 times) from 0% to 100% strain at a rate of 10 mm/min. The Young’s moduli (*E*, kPa) and toughness (*U_T_*, kJ/m^3^) were calculated using the following equations:

*E* = *σ*/*ε* (1)

(2) $$U_{T}=\int_{0}^{\varepsilon }\sigma d\varepsilon$$

where *σ* is the uniaxial stress (*N*/m^2^) and *ε* is the uniaxial strain.

### 2.4. Mechanical and Rheological Characterization of the Alg, CMC, and PAAm-Based Conductive Hydrogels

The rheological behavior of the STICH and the controls were investigated using a Discovery Hybrid Rheometer 2 (TA Instruments, New Castle, DE, USA) in the oscillation-frequency-sweep mode. The samples used in these experiments were prepared using manually fabricated cylindrical molds (12.5 × 12.5 × 1 mm^3^). All rheological measurements were performed using parallel-plate geometry (diameter: 20 mm). The oscillation-frequency-sweep measurements were used to evaluate the variation in storage (*G*′) and loss moduli (*G*”) with the frequency from 0.01 Hz to 10 Hz. The geometric gap and axial force were fixed at 1 mm and 0.1 N, respectively, during the rheological measurements. The storage modulus (*G*’), loss modulus (*G*”), energy dissipation value, tan delta (*tan* δ), and frequency-dependent complex modulus (G*) were calculated using the following equations [84]:

*ε* = *ε*_0_ sin (*ωt*), *σ* = *σ*_0_ sin (*ωt* + *δ*) (3)

*G*’ = *σ*_0_/*ε*_0_ (cos *δ*) (4)

*G*” = *σ*_0_/*ε*_0_ (sin *δ*) (5)

*Tan δ* = *G*”/*G*’ (6)

*Dynamic modulus* (G*) = (*G*’^2^ + *G*”^2^)^1/2^ (7)

where *σ* is the stress (Pa), *ε* is the strain (mm/mm), *ω* is the frequency of strain oscillation (rad[°]/s), *t* is the time (s), and *δ* is the phase lag between stress and strain (rad[°]/s)

### 2.5. Electrical Characterization under Strain of the Stretchable Surface Electrode Arrays

Stretching tests were performed to characterize the electrical performance of the stretchable surface electrodes under strain using the following protocol: A piece of a polyethylene terephthalate film was attached to the auto-stretching stage (Motorizer X-translation Stages, Jaeil Optical System) to hold both edges of the stretchable device patch. The sample was loaded onto the stretching stage and robustly fixed using tape (3M Co., Ltd.) and silicon epoxy (Sil-poxy, Smooth-On Co., Ltd.). A droplet of liquid metal (gallium indium eutectic, Sigma Aldrich) was applied to the electrode channel and contact pad, followed by wiring cables up to a measurement instrument. A source meter (Keithley 2450 SourceMeter, TEKTRONIX, Inc.) was used to measure the electrical performance of the stretchable electrodes. The resistance of the stretchable electrode was measured according to the stretching range using LabVIEW customized software (National Instrument Corp.).

### 2.6. Electrochemical Impedance of the Surface Electrode Arrays Using the STICH

Electrochemical impedance spectroscopy (EIS) was performed using a potentiostat (SP1, ZIVE Lab Co., Ltd.) to characterize the electrochemical impedance properties of the stretchable surface electrodes. A three-electrode configuration was used to measure the device impedance. A saturated calomel electrode (BASiAg/AgCl/3 M NaCl) and Pt plate (RDE0021, AT Frontier Co., Ltd.) were used as the reference and counter electrodes, respectively. Using a root-mean-square voltage of 10 mV as the input source of the potentiostatic EIS measurements, impedance plots of the electrodes were acquired over a frequency range of 100 kHz–10 Hz using phosphate-buffered saline (PBS; Samshun Pure Chemical Co., Ltd.). To evaluate the possible improvement in electrochemical impedance induced by the STICH, impedance plots were acquired for the same electrode before and after the application of the STICH coating.

### 2.7. Acute in Vivo ECoG Recording and Visual Evoked Potential (VEP) Activation Tests

Animal experiments for in vivo ECoG monitoring and VEP activation were conducted on anesthetized rodents (Sprague–Dawley rats, 8 weeks old). For the acute tests, the animals were anesthetized via the intramuscular injection of a ketamine/xylazine mixture, and the head of the animal was fixed on a stereotaxic instrument (Model 900, David Kopf Instrument, Inc.). The scalp of the anesthetized animal was incised, and the skull was removed using an electric drill (Strong 207s, Saeshin, Inc.) to secure the cerebral cortex in the left hemisphere. The stretchable surface electrode arrays were connected to a neural recording instrument (Digital Lynx SX, Neuralynx, Inc.) using an assembled, customized adaptor. The device was mounted on the cerebral cortex, and baseline ECoG signal recordings were performed for 5 min. After the baseline signal recordings, the anesthetized animals were subjected to dark adaptation for approximately 5 min, followed by a VEP activation test for 5 min. To achieve VEP activation in the left hemisphere, light stimulation was conducted at a frequency of 0.2 Hz with respect to the right eye of the animal using a closely attached green LED while its left eye was covered. To evaluate the contribution of the conductive hydrogel to the signal acquisition performance, the baseline signal recording and VEP activation tests were sequentially performed on the same electrode device before and after the hydrogel coating. The documented ECoG data were processed and profiled using MATLAB.

## 3. Results and Discussion

### 3.1. Physicochemical Properties of the STICH

**Figure 2:**
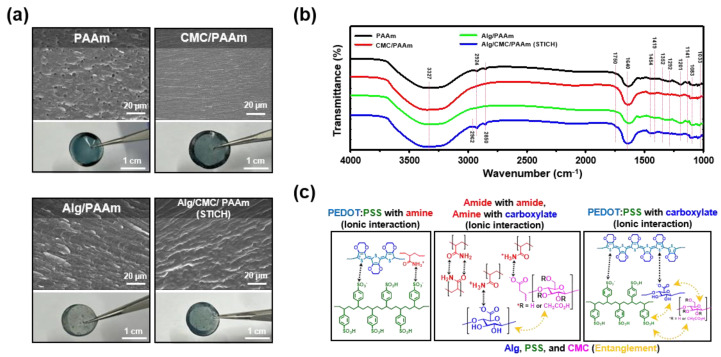
Physicochemical characterization of the Alg, CMC, and PAAm-based conductive hydrogels. (**a**) Cross-sectional SEM images (top, scale bar = 20 µm) and photographs (bottom, scale bar = 1 cm) of each Alg, CMC, and PAAm-based conductive hydrogel. (**b**) FT-IR spectra of the Alg, CMC, and PAAm-based conductive hydrogels. (**c**) Mechanism of the ionic interactions between the PEDOT:PSS/PAAm, PAAm/polyanions (Alg or CMC), and PEDOT:PSS/Alg. (“PAAm”, black; “Alg/PAAm”, green; “CMC/PAAm”, red; and “Alg/CMC/PAAm”, the STICH, blue).

Cross-sectional SEM images and photographs (Figure 2a, top, and bottom, respectively) confirmed the morphological stability of the STICH and other control materials of the Alg, CMC, and PAAm-based conductive hydrogels fabricated according to the conditions in Table 1. Overall, the composite hydrogels showed a nonporous structure (except PAAm) owing to the intense interactions between the backbones (particularly between PEDOT:PSS and the polyelectrolytes). Additionally, as the Alg content (wt.%) increased, the strong ionic interactions between the PEDOT:PSS and carboxylate increased with the transparency of the STICH; however, a large amount of PEDOT:PSS precipitates was present in the gels (Figure 2a, third box). Nevertheless, the pristine CMC and the Alg/CMC mixture exhibited reduced aggregation, suggesting that the CMC presumably interrupted the ionic interactions between Alg and PAAm.

The FT-IR peaks provided clear evidence for the aforementioned hypothesis (Figure 2b). The broad peaks from 3000 to 3600 cm^−1^ correspond to the stretching vibrations of the N–H, O–H, ether, and carboxylic functional groups [85,86]. The three peaks at approximately 1419, 1352, and 1049 cm^−1^ were assigned to the stretching vibration of the S=O bond in the PSS chain [87,88]. The other three peaks at 1201, 1141, and 1083 cm^−1^ were assigned to the stretching vibrations of C–O. The peaks at approximately 1750, 1640, and 1292 cm^−1^ were assigned to the “stretching vibration of C=O” and the “bending vibrations of N–H, (C=O)NH_2_ and C–N”. Finally, the three small peaks at 2962, 2924, and 2850 cm^−1^ were assigned to the stretching vibrations of C–H_2_, C–H_3_, and C=O, respectively. Compared to the FT-IR spectra of the “Alg/PAAm” and “CMC/PAAm”, that of the “Alg/CMC/PAAm/PEDOT:PSS” (STICH) showed three peaks (C–H_2_, C–H_3_, and C=O), which could be attributed to the disruption of the ionic interactions between the amide and carboxylate. In summary, the STICH specimen that had a compact entangled network (e.g., the complex between Alg, CMC, and PSS) and various ionic interactions between polyanions (Alg, CMC, PSS) and polycations (PEDOT, PAAm) induced both ionic interactions and dimensional interruptions in the system, which ruptured the binding and increased the durability (Figure 2c).

### 3.2. Mechanical Properties of the STICH

**Figure 3:**
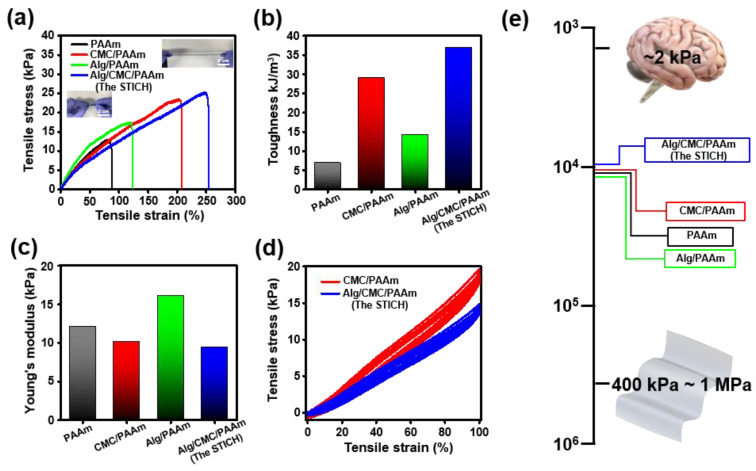
Tensile strength measurements of the Alg, CMC, and PAAm-based conductive hydrogels. (**a**) The graph shows the tensile stress-strain curves (inset image scale bar = 2 cm) of the Alg, CMC, and PAAm-based conductive hydrogels (*n* = 3). (**b**) Toughness of the Alg, CMC, and PAAm-based conductive hydrogels (**c**) Young’s moduli of the Alg, CMC, and PAAm-based conductive hydrogels. (**d**) Cyclic stretching data (10 times) of “CMC/PAAm” and STICH. (**e**) Young’s moduli on the log scale of the Alg, CMC, and PAAm-based conductive hydrogels, brain, and PDMS. (“PAAm”, black; “Alg/PAAm”, green; “CMC/PAAm”, red; and “Alg/CMC/PAAm”, the STICH, blue).

The results of the mechanical property characterization also supported our hypothesis based on the aforementioned physicochemical characterization (Figure 3a–d). The UTM measurements of the Alg, CMC, and PAAm-based conductive hydrogels were performed using our previously reported protocol to evaluate the maximum tensile strain per stress of each Alg, CMC, and PAAm-based conductive hydrogel with varying toughness values and Young’s moduli [89]. Notably, the single-network system of conductive hydrogels (such as “PAAm”) could not endure a strain of more than 100%. However, the additional polyanion backbones strengthened the conductive hydrogel system, with the triple-network (such as “STICH”) enduring a strain of more than 250% (Figure 3a) with high toughness (36.85 kJ/m^3^; Figure 3b) but a low Young’s modulus (9.46 kPa; Figure 3c). On the other hand, in contrast to the single-network system of a conductive hydrogel, the elongation ratio in the triple-network system containing different kinds of polyanion volumes was significantly increased by three-fold while Young’s modulus was decreased because of the CMC-originated energy relaxation (based on the presumable interruption of ionic interactions between Alg and PAAm). The cyclic stretching tests indicated that the STICH exhibited a lower Young’s modulus without residual strain than the dual-network gel system (such as “CMC/PAAm”) (Figure 3d). Normally, brain-targeted bioelectronics requires a low Young’s modulus (~100 kPa) for modulus matching with brain tissue (~1 kPa) but high toughness to support the upper elastic substrate of the bioelectronic (such as PDMS). Therefore, the STICH is a particularly appropriate hydrogel platform for bioelectronics compared to the other investigated hydrogel systems (Figure 3e). 

### 3.3. Rheological Properties of the STICH

**Figure 4:**
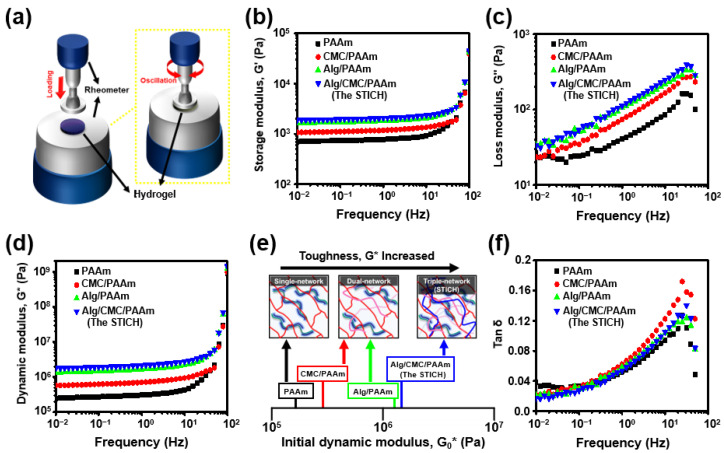
Rheological characterization of the Alg, CMC, and PAAm-based conductive hydrogels. (**a**) The experimental setting illustration of the rheological property measurements of the Alg, CMC, and PAAm-based conductive hydrogels (*n* = 3). (**b**) Storage modulus (*G*’) of each Alg, CMC, and PAAm-based conductive hydrogel. (**c**) Loss modulus (*G*”) of each Alg, CMC, and PAAm-based conductive hydrogel. (**d**) Dynamic modulus (G*) of each Alg, CMC, and PAAm-based conductive hydrogel. (**e**) Initial dynamic modulus on a log scale for “PAAm” (single-network), “CMC/PAAm” and “Alg/PAAm” (dual-network), and STICH (triple-network). (**f**) *Tan* δ of each Alg, CMC, and PAAm-based conductive hydrogel. (“PAAm”, black; “Alg/PAAm”, green; “CMC/PAAm”, red; and “Alg/CMC/PAAm”, the STICH, blue).

Normally, rheological behaviors that could be determined by analyzing the oscillatory behaviors of viscoelastic materials in parallel-plate geometry were typically evaluated by varying the frequency and monitoring through the absolute values of the storage modulus (*G’*), loss modulus (*G*”), and absolute rate distinctions between *G’* and *G*” (nominated as *tan* δ). The dynamic mechanical properties of the Alg, CMC, and PAAm-based conductive hydrogels were easily calculated using Equations (3)–(6). The schematic in Figure 4a illustrates the principle of the rheological experiments; the *G’* and *G*” data are shown in Figure 4b and 4c, respectively. The oscillation frequency sweep results of *G’*, *G*”, *tan* δ, and G* ultimately supported our hypothesis based on the aforementioned Alg, CMC, and PAAm-based conductive hydrogel characterization (Figure 4a–f). The tendencies of the oscillation results were mostly the same as the mechanical test results, which exhibited higher moduli than those of the single (such as “PAAm”), dual, (such as “CMC/PAAm” and “Alg/PAAm”), and triple-networks (STICH) (Figure 4b,c). Especially, the frequency dependence result of G* showed that the storage modulus dominates all of the viscoelastic responses from the whole frequency range (Figure 4d). Furthermore, the “PAAm” (single-network), “CMC/PAAm” and “Alg/PAAm” (dual-networks), and STICH (triple-network) comparison graph of the initial dynamic moduli clearly demonstrated the correlation between the frequency dependence and tensile deformation rate, and the complex network system increased the whole elasticity criterion. In contrast, the *tan* δ results showed that STICH exhibited similar results to the single-network hydrogel, evidently dissipating the dynamic stress effectively (Figure 4f). In summary, our STICH is the most optimum Alg, CMC, and PAAm-based conductive hydrogel to facilitate soft bioelectronic applications, the softest hydrogel platform compared with single and dual-networks.

### 3.4. Electrical Performance of the Stretchable Electrode and the Electrochemical Impedance of the Device Using the STICH

**Figure 5:**
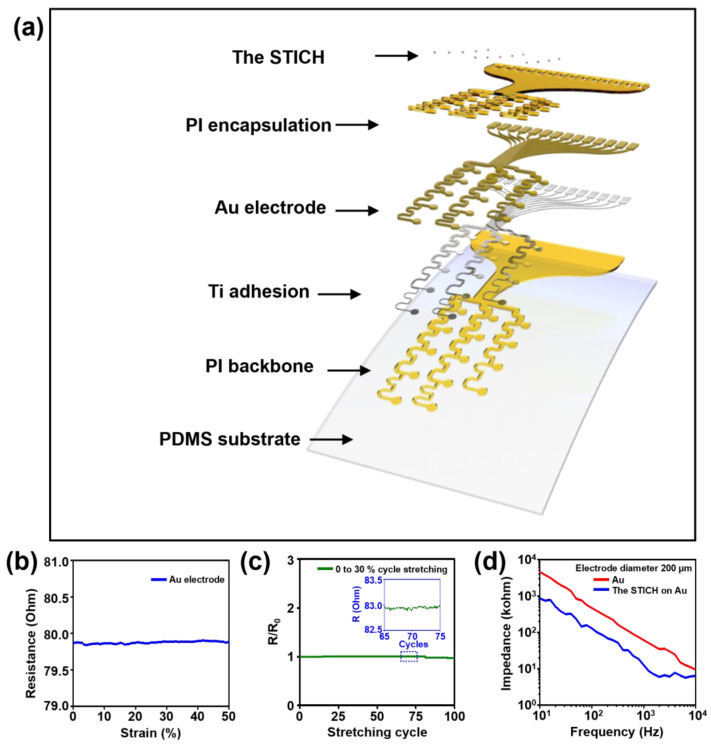
Electrical characteristics of the stretchable surface electrode arrays and the in vitro EIS analysis of the STICH-coated devices. (**a**) Exploded-view schematic of the stretchable surface electrode arrays. (**b**) Electrical resistance of the stretchable surface electrode while stretching up to 50% strain. (**c**) Normalized electrical resistance during 100 stretching cycles up to 30% strain (inset: a representative plot of 10 cycles). (**d**) EIS plots of the Au electrode (red line) and the STICH-coated electrode (blue) with respect to frequency (from 100 kHz to 10 Hz).

The multi-channel (12-channel for sensing, a reference, and a ground channel) surface electrode array devices were finely manufactured via a microfabrication process (Figure 5a). The resistance of the stretchable Au electrode with a 200 μm diameter was approximately 80 Ω. The wavy, serpentine pattern of the lateral interconnects provided stretchability for the thin-film devices without any degradation of electrical characteristics up to 50% maximum strain, which was sufficient to achieve adaptive integration with the curved cerebral cortex (Figure 5b). This structural design exhibited mechanical durability in terms of maintaining a constant electrical performance during cyclic stretching at 30% strain (Figure 5c). The mechanical deformability and durability of our electrode design are enough to be used in brain interfacing devices. The electrochemical impedance of an Au electrode with a 200 μm diameter) was marked at approximately 60 kΩ at 1 kHz. The impedance plot of the Au electrode increased linearly up to approximately 5 MΩ at 10 Hz (Figure 5d, red line). In comparison, the impedance corresponding to the frequency band in the same electrode channel with the STICH coating considerably decreased, particularly in the low-frequency range (Figure 5d, blue line). The impedance of the STICH-coated electrode at 1 kHz was reduced to approximately 10 kΩ, and the maximum value at 10 Hz was also decreased to 860 kΩ. This reduction in impedance across the frequency band means that the electrophysiology recording capabilities of the electrode devices were further improved, so it could be expected that a higher sensitive recording of brain activity would be achieved during ECoG usage. These results demonstrated the STICH-induced improvements in the electrochemical characteristics, particularly with respect to electrophysiological performance.

### 3.5. In Vivo Experiment Results of Acute ECoG Monitoring

**Figure 6:**
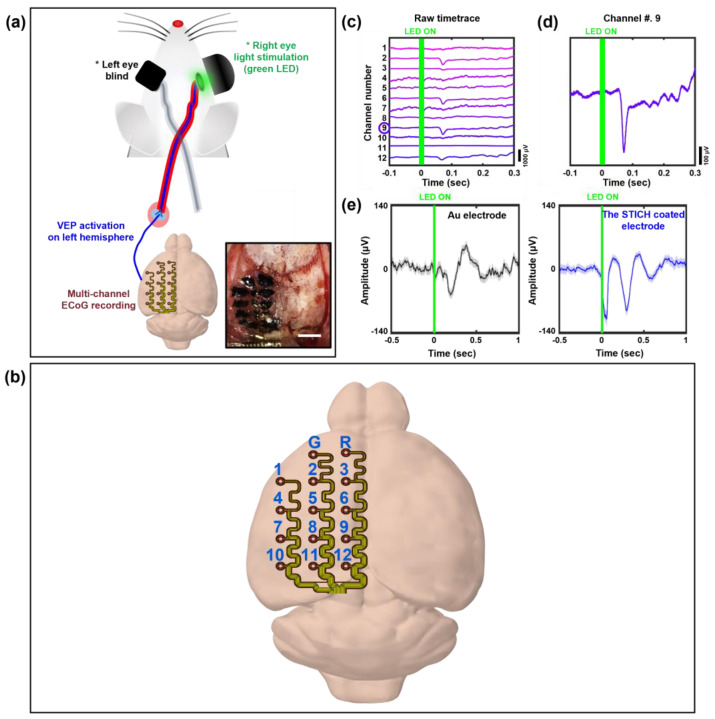
Experimental results of the acute in vivo ECoG monitoring and VEP activation tests conducted using an anesthetized rodent model. (**a**) Schematic of ECoG monitoring during VEP activation on the left hemisphere of a rat by stimulating its right eye using a green LED (inset: a photograph of the STICH-coated stretchable surface electrode arrays mounted on the left hemispheric cortex, the scale bar indicates 3 mm). (**b**) Two-dimensional deployment map of the multi-channel ECoG electrodes mounted on the rodent’s cortex. (**c**) Representative raw plot of the baseline ECoG and VEP signals recorded using the multi-channel surface electrode arrays. (**d**) An activated VEP signal recorded from a representative channel. (**e**) Average VEP plot derived from 140 trials of light stimulation tests. The difference in amplitude of the average VEPs before (left) and after (right) the STICH coating demonstrates the significant contribution of the STICH in improving the neural recording performance of the electrodes.

To demonstrate the feasibility of the STICH for brain-interfacing applications, in vivo experiments for ECoG monitoring were conducted using the stretchable electrode arrays coated with the STICH brain-interfacing layer. In the acute experiments, an electrode array device with the STICH layer was attached to the left hemisphere of an anesthetized rat, and the baseline ECoG signals and event-related potentials (ERPs) from the visual cortex (VEP) activated by light stimulation of the right eye were recorded (Figure 6a). The direct contact between the STICH layers with the cortex permitted the construction of adaptive brain interfacing. The brain-like softness of the STICH provided excellent conformality to electrodes, and the sufficient toughness allowed the STICH layer to maintain its solid form between the device and the tissue. The raw signal plot of the baseline ECoG activity from the surface electrodes mounted on the animal’s cortex (Figure 6b) and activated VEP emphasized the selectivity of the 12-channel STICH-based electrode arrays (Figure 6c). Moreover, the STICH-coated electrodes featured the capability of clearly recording visual cortex-originated ERPs (Figure 6d). The contribution of the STICH was intuitively demonstrated by comparing the average amplitude of multiple activated VEPs recorded from the electrode using the STICH with that from the control device without the STICH coating (Figure 6e). The average amplitude of multiple VEPs (a total of 140 accumulated trials) from the STICH electrodes was significantly larger (~133 μV) than that from the control device (~109 μV). As expected, the improvement of electrochemical impedance performance by the use of the STICH coating resulted in a higher sensitive recording of ECoG activity in vivo. These results demonstrate the STICH-induced improvements in the neural recording performance of the electrodes in terms of both electrical and ionic conductivities.

## 4. Conclusions

In this study, a new functional composite hydrogel, the STICH, was developed to construct an adaptive conformal brain interface. The triple-network-based STICH exhibited both high toughness and a low Young’s modulus, which was ideal in terms of applicability to soft brain tissue. This mechanical improvement originated from the dissipated hydrogen bonding and entanglement between Alg, CMC, and PAAm. Furthermore, the STICH, with both electrical and ionic conductivities, bestowed improved electrochemical impedance properties to metal electrodes upon coating. Consequently, adaptive conformal brain interfacing and capable ECoG monitoring were successfully achieved in a rodent model using the stretchable surface electrode arrays with the STICH. Based on the improvement in electrochemical impedance, the electrode device with the STICH layer exhibited a more sensitive electrophysiology recording capability with a higher amplitude of visual ERPs than that of the control device. The implementation of these functionalities, which could be realized for translational applications, could permit the development of advanced bioelectronic materials and devices that are more practical and applicable in clinical settings.

## Figures and Tables

**Table 1:** The precursor compositions of different Alg, CMC, and PAAm-based conductive hydrogels.

Sample Name	Di Water (g)	Alg (g)	AAm (g)	CMC (g)	Clevios™ PH 1000 (PEDOT:PSS Dispersion) (g)	MBAA (g)
PAAm	5	-	0.8	-	1	0.0005
Alg/PAAm	5	0.1	0.8	-	1	0.0005
CMC/PAAm	5	-	0.8	0.1	1	0.0005
STICH (Alg/CMC/PAAm)	5	0.05	0.8	0.05	1	0.0005

Alg = sodium alginate; AAm = acrylamide; CMC = carboxymethyl cellulose; Clevios™ PH 1000 = commercial PEDOT/PSS solution product; MBAA = N N′ methylenebisacrylamide.

## Data Availability

Not applicable.

## Institutional Animal Care and Use Committee

The animal experiments for ECoG monitoring were conducted by obtaining Institutional animal care and use committee (IACUC) approval (SKKU IACUC2022-08-23-1) from Sungkyunkwan University.

## References

[1] Thakor N.V. (2013). Translating the Brain-Machine Interface. Sci. Transl. Med..

[2] Kim C.K., Yang S.J., Pichamoorthy N., Young N.P., Kauvar I., Jennings J.H., Deisseroth K., Lerner T.N., Berndt A., Lee S.Y. (2016). Simultaneous fast measurement of circuit dynamics at multiple sites across the mammalian brain. Nat. Methods.

[3] Stirman J., Smith I., Kudenov M., Simth S.L. (2016). Wide field-of-view, multi-region, two-photon imaging of neuronal activity in the mammalian brain. Nat. Biotechnol..

[4] Liu J., Fu T.-M., Cheng Z., Hong G., Zhou T., Jin L., Duvvuri M., Jiang Z., Kruskal P., Lieber C.M. (2015). Syringe-injectable electronics. Nat. Nanotech..

[5] Xie C., Liu J., Fu T.-M., Dai X., Zhou W., Lieber C.M. (2015). Three-dimensional macroporous nanoelectronic networks as minimally invasive brain probes. Nat. Mater..

[6] Park S., Guo Y., Jia X., Choe H.K., Grena B., Kang J., Park J., Lu C., Canales A., Anikeeva P. (2017). One-step optogenetics with multifunctional flexible polymer fibers. Nat. Neurosci..

[7] Canales A., Jia X., Froriep U.P., Koppes R.A., Tringides C.M., Selvidge J., Lu C., Hou C., Wei L., Anikeeva P. (2015). Multifunctional fibers for simultaneous optical, electrical and chemical interrogation of neural circuits in vivo. Nat. Biotechnol..

[8] Zhang Z., Russell L.E., Packer A.M., Gauld O.M., Häusser M. (2018). Closed-loop all-optical interrogation of neural circuits in vivo. Nat. Methods.

[9] Park S.I., Brenner D.S., Shin G., Morgan C.D., Copits B.A., Chung H.U., Pullen M.Y., Noh K.N., Davidson S., Rogers J.A. (2015). Soft, stretchable, fully implantable miniaturized optoelectronic systems for wireless optogenetics. Nat. Biotechnol..

[10] Krauss J.K., Lipsman N., Aziz T., Boutet A., Brown P., Chang J.W., Davidson P., Grill W.M., Haria M.I., Lozano A.M. (2021). Technology of deep brain stimulation: Current status and future directions. Nat. Rev. Neurol..

[11] Hong G., Lieber C.M. (2019). Novel electrode technologies for neural recordings. Nat. Rev. Neurosci..

[12] Qazi R., Gomez A.M., Castro D.C., Zou Z., Sim J.Y., Xiong Y., Abdo J., Kin C.Y., Anderson A., Jeong J.-W. (2019). Wireless optofluidic brain probes for chronic neuropharmacology and photostimulation. Nat. Biomed. Eng..

[13] Huang Y., Cui Y., Deng H., Wang J., Hong R., Hu S., Hou H., Dong Y., Wang H., Sheng X. (2022). Bioresorbable thin-film silicon diodes for the optoelectronic excitation and inhibition of neural activities. Nat. Biomed. Eng.

[14] Bansal A., Shikha S., Zhang Y. (2022). Towards translational optogenetics. Nat. Biomed. Eng..

[15] Mohanty A., Li Q., Tadayon M.A., Roberts S.P., Bhatt G.R., Shim E., Ji X., Cardens J., Miller S.A., Lipson M. (2020). Reconfigurable nanophotonic silicon probes for sub-millisecond deep-brain optical stimulation. Nat. Biomed. Eng..

[16] Cagnan H., Denison T., McIntyre C., Brown P. (2019). Emerging technologies for improved deep brain stimulation. Nat. Biotechnol..

[17] Kim T., McCall J.G., Jung Y.H., Huang X., Siuda E.R., Li Y., Song J., Song Y.M., Pao H.A., Bruchas M.R. (2013). Injectable, Cellular-Scale Optoelectronics with Applications for Wireless Optogenetics. Science.

[18] Patel S.R., Lieber C.M. (2019). Precision electronic medicine in the brain. Nat. Biotechnol..

[19] Lacour S., Courtine G., Guck J. (2016). Materials and technologies for soft implantable neuroprostheses. Nat. Rev. Mater..

[20] Chen R., Canales A., Anikeeva P. (2017). Neural recording and modulation technologies. Nat. Rev. Mater..

[21] Normann R. (2007). Technology Insight: Future neuroprosthetic therapies for disorders of the nervous system. Nat. Rev. Neurol..

[22] Jackson A., Zimmermann J. (2012). Neural interfaces for the brain and spinal cord—Restoring motor function. Nat. Rev. Neurol..

[23] Bensmaia S., Miller L. (2014). Restoring sensorimotor function through intracortical interfaces: Progress and looming challenges. Nat. Rev. Neurosci..

[24] Feiner R., Dvir T. (2018). Tissue–electronics interfaces: From implantable devices to engineered tissues. Nat. Rev. Mater..

[25] Frank J.A., Antonini M.J., Anikeeva P. (2019). Next-generation interfaces for studying neural function. Nat. Biotechnol..

[26] Cehajic-Kapetanovic J., Singh M.S., Zrenner E., MacLaren R.E. (2022). Bioengineering strategies for restoring vision. Nat. Biomed. Eng..

[27] Viventi J., Kim D.-H., Vigeland L., Frechette E.S., Blanco J.A., Kim Y.-S., Avrin A.E., Tiruvadi V.R., Hwang S.-W., Litt B. (2011). Flexible, foldable, actively multiplexed, high-density electrode array for mapping brain activity in vivo. Nat. Neurosci..

[28] Yu K.J., Kuzum D., Hwang S.-W., Kim B.H., Juul H., Kim N.H., Won S.M., Chiang K., Trumpis M., Rogers J.A. (2016). Bioresorbable silicon electronics for transient spatiotemporal mapping of electrical activity from the cerebral cortex. Nat. Mater..

[29] Chiang C.-H., Won S.M., Orsborn A.L., Yu K.J., Trumpis M., Bent B., Wang C., Xue Y., Min S., Viventi J. (2020). Development of a neural interface for high-definition, long-term recording in rodents and nonhuman primates. Sci. Transl. Med..

[30] Khodagholy D., Gelinas J.N., Thesen T., Doyle W., Devinsky O., Malliaras G.G., Buzsáki G. (2015). NeuroGrid: Recording action potentials from the surface of the brain. Nat. Neurosci..

[31] Tchoe Y., Bourhis A.M., Cleary D.R., Stedelin B., Lee J., Tonsfeldt K.J., Brown E.C., Siler D.A., Paulk A.C., Dayeh S.A. (2022). Human brain mapping with multithousand-channel PtNRGrids resolves spatiotemporal dynamics. Sci. Transl. Med..

[32] Masvidal-Codina E., Illa X., Dasilva M., Calia A.B., Dragojević T., Vidal-Rosas E.E., Prats-Alfonso E., Gldigon P., Rius G., Guimerà-Brunet A. (2019). High-resolution mapping of infraslow cortical brain activity enabled by graphene microtransistors. Nat. Mater..

[33] Bonaccini Calia A., Masvidal-Codina E., Smith T.M., Schäfer N., Rathore D., Rodríguez-Lucas E., Illa X., Corro E.D., Viana D., Garrido J.A. (2022). Full-bandwidth electrophysiology of seizures and epileptiform activity enabled by flexible graphene microtransistor depth neural probes. Nat. Nanotechnol..

[34] Zhao Z., Zhu H., Li X., Sun L., He F., Chung J.E., Liu D.F., Frank L., Luan L., Xie C. (2022). Ultraflexible electrode arrays for months-long high-density electrophysiological mapping of thousands of neurons in rodents. Nat. Biomed. Eng..

[35] Jun J.J., Steinmetz N.A., Siegle J.H., Denman D.J., Bauza M., Barbarits B., Lee A.K., Anastassiou C.A., Andrei A., Harris T.D. (2017). Fully integrated silicon probes for high-density recording of neural activity. Nature.

[36] Steinmetz N.A., Aydin C., Lebedeva A., Okun M., Pachitariu M., Bauza M., Beau M., Bhagat J., Broux M., Harris T.D. (2021). Neuropixels 2.0: A miniaturized high-density probe for stable, long-term brain recordings. Science.

[37] Paulk A.C., Kfir Y., Khanna A.R., Mustroph M.L., Trautmann E.M., Soper D.J., Stavisky S.D., Welkenhuysen M., Dutta B., Cash S.S. (2022). Large-scale neural recordings with single neuron resolution using Neuropixels probes in human cortex. Nat. Neurosci..

[38] Kathe C., Michoud F., Schönle P., Rowald A., Brun N., Ravier J., Furfaro I., Paggi V., Kim K., Courtine G. (2022). Wireless closed-loop optogenetics across the entire dorsoventral spinal cord in mice. Nat. Biotechnol..

[39] Szuts T.A., Fadeyev V., Kachiguine S., Sher A., Grivich M.V., Agrochão M., Hottowy P., Dabrowski W., Lubenov E.V., Meister M. (2011). A wireless multi-channel neural amplifier for freely moving animals. Nat. Neurosci..

[40] Schwarz D.A., Lebedev M.A., Hanson T.L., Dimitrov D.F., Lehew G., Meloy J., Rajangam S., Subramanian V., Ifft P.J., Nicolelis M.A.L. (2014). Chronic, wireless recordings of large-scale brain activity in freely moving rhesus monkeys. Nat. Methods.

[41] Montgomery K.L., Yeh A.J., Ho J.S., Tsao V., Mohan Iyer S., Grosenick L., Ferenczi E.A., Tanabe Y., Deisseroth K., Poon A.S.Y. (2015). Wirelessly powered, fully internal optogenetics for brain, spinal and peripheral circuits in mice. Nat. Methods.

[42] Even-Chen N., Muratore D.G., Stavisky S.D., Hochberg L.R., Henderson J.M., Murmann B., Shenoy K.V. (2020). Power-saving design opportunities for wireless intracortical brain–computer interfaces. Nat. Biomed. Eng..

[43] Lee J., Leung V., Lee A.-H., Huang J., Asbeck P., Mercier P.P., Shellhammer S., Larson L., Laiwall F., Nurmikko A. (2021). Neural recording and stimulation using wireless networks of microimplants. Nat. Electron..

[44] Borton D., Micera S., Millán J.d.R., Courtine G. (2013). Personalized Neuroprosthetics. Sci. Transl. Med..

[45] Bonizzato M., Martinez M. (2021). An intracortical neuroprosthesis immediately alleviates walking deficits and improves recovery of leg control after spinal cord injury. Sci. Transl. Med..

[46] Capogrosso M., Milekovic T., Borton D., Wagner F., Moraud E.M., Mignardot J.-B., Buse N., Gandar J., Barraud Q., Courtine G. (2016). A brain–spine interface alleviating gait deficits after spinal cord injury in primates. Nature.

[47] Michoud F., Seehus C., Schönle P., Brun N., Taub D., Zhang Z., Jain A., Furfaro I., Akouissi O., Lacour S.P. (2021). Epineural optogenetic activation of nociceptors initiates and amplifies inflammation. Nat. Biotechnol..

[48] Nelson A., Abdelmesih B., Costa R.M. (2021). Corticospinal populations broadcast complex motor signals to coordinated spinal and striatal circuits. Nat. Neurosci..

[49] Gutruf P., Krishnamurthi V., Vázquez-Guardado A., Xie Z., Banks A., Su C.-J., Xu Y., Hanry C.R., Water E.A., Rogers J.A. (2018). Fully implantable optoelectronic systems for battery-free, multimodal operation in neuroscience research. Nat. Electron..

[50] Vázquez-Guardado A., Yang Y., Bandodkar A.J., Rogers J.A. (2020). Recent advances in neurotechnologies with broad potential for neuroscience research. Nat. Neurosci..

[51] Yang Y., Wu M., Vázquez-Guardado A., Wegener A.J., Grajales-Reyes J.G., Deng Y., Wang T., Avila R., Moreno J.A., Rogers J.A. (2021). Wireless multilateral devices for optogenetic studies of individual and social behaviors. Nat. Neurosci..

[52] Qazi R., Parker K.E., Kim C.Y., Rill R., Norris M.R., Chung J., Bilbily J., Kim J.R., Walicki M.C., Jeong J.-W. (2022). Scalable and modular wireless-network infrastructure for large-scale behavioural neuroscience. Nat. Biomed. Eng..

[53] Stringer C., Pachitariu M., Steinmetz N., Reddy C.B., Carandini M., Harris K.D. (2019). Spontaneous behaviors drive multidimensional, brainwide activity. Science.

[54] Gelinas J.N., Khodagholy D., Thesen T., Devinsky O., Buzsáki G. (2016). Interictal epileptiform discharges induce hippocampal–cortical coupling in temporal lobe epilepsy. Nat. Med..

[55] Khodagholy D., Gelinas J.N., Buzsáki G. (2017). Learning-enhanced coupling between ripple oscillations in association cortices and hippocampus. Science.

[56] Fernández-Ruiz A., Oliva A., Soula M., Rocha-Almeida F., Nagy G.A., Martin-Vazquez G., Buzsáki G. (2021). Gamma rhythm communication between entorhinal cortex and dentate gyrus neuronal assemblies. Science.

[57] Tian L., Zimmerman B., Akhtar A., Yu K.J., Moore M., Wu J., Larsen R.J., Lee J.W., Li J., Rogers J.A. (2019). Large-area MRI-compatible epidermal electronic interfaces for prosthetic control and cognitive monitoring. Nat. Biomed. Eng..

[58] Rossant C., Kadir S.N., Goodman D.F.M., Schulman J., Hunter M.L.D., Saleem A.B., Grosmark A., Belluscio M., Denfiels G.H., Ecker A.S. (2016). Spike sorting for large, dense electrode arrays. Nat. Neurosci..

[59] Fu T.-M., Hong G., Zhou T., Schuhmann T.G., Viveros R.D., Lieber C.M. (2016). Stable long-term chronic brain mapping at the single-neuron level. Nat. Methods.

[60] Nason S.R., Vaskov A.K., Willsey M.S., Welle E.J., An H., Vu P.P., Bullard A.J., Nu C.S., Kao J.C., Chestek C.A. (2020). A low-power band of neuronal spiking activity dominated by local single units improves the performance of brain–machine interfaces. Nat. Biomed. Eng..

[61] Cea C., Spyropoulos G.D., Jastrzebska-Perfect P., Ferrero J.J., Gelinas J.N., Khodagholy D. (2020). Enhancement-mode ion-based transistor as a comprehensive interface and real-time processing unit for in vivo electrophysiology. Nat. Mater..

[62] Kim D.-H., Viventi J., Amsden J.J., Xiao J., Vigeland L., Kim Y.-S., Blanco J.A., Panilaitis B., Frechette E.S., Rogers J.A. (2010). Dissolvable films of silk fibroin for ultrathin conformal bio-integrated electronics. Nat. Mater..

[63] Salatino J.W., Ludwig K.A., Kozai T.D.Y., Purcell E.K. (2017). Glial responses to implanted electrodes in the brain. Nat. Biomed. Eng..

[64] Song E., Li J., Won S.M., Bai W., Rogers J.A. (2020). Materials for flexible bioelectronic systems as chronic neural interfaces. Nat. Mater..

[65] Li J., Liu Y., Yuan L., Zhang B., Bishop E.S., Wang K., Tang J., Zheng Y.-Q., Xu W., Bao Z. (2022). A tissue-like neurotransmitter sensor for the brain and gut. Nature.

[66] Yang Q., Wei T., Yin R.T., Wu M., Xu Y., Koo J., Choi Y.S., Xie Z., Chen S.W., Rogers J.A. (2021). Photocurable bioresorbable adhesives as functional interfaces between flexible bioelectronic devices and soft biological tissues. Nat. Mater..

[67] Tringides C.M., Vachicouras N., de Lázaro I., Wang H., Trouillet A., Seo B.R., Fallegger F., Shin Y., Casiraghi C., Mooney D.J. (2021). Viscoelastic surface electrode arrays to interface with viscoelastic tissues. Nat. Nanotechnol..

[68] Oribe S., Yoshida S., Kusama S., Osawa S., Nakagawa A., Iwasaki M., Tominaga T., Nishizawa M. (2019). Hydrogel-Based Organic Subdural Electrode with High Conformability to Brain Surface. Sci. Rep..

[69] Feig V.R., Tran H., Lee M., Bao Z. (2018). Mechanically Tunable Conductive Interpenetrating Network Hydrogels That Mimic the Elastic Moduli of Biological Tissue. Nat. Commun..

[70] Liu Y., Liu J., Chen S., Lei T., Kim Y., Niu S., Wang H., Wang X., Foudeh A.M., Tok J.B.-H. (2019). Soft and Elastic Hydrogel-Based Microelectronics for Localized Low-Voltage Neuromodulation. Nat. Biomed. Eng..

[71] Cho Y.U., Lim S.L., Hong J.-H., Yu K.J. (2022). Transparent Neural Implantable Devices: A Comprehensive Review of Challenges and Progress. Npj Flex. Electron..

[72] Kim Y., Song J., An S., Shin M., Son D. (2022). Soft Liquid Metal-Based Conducting Composite with Robust Electrical Durability for a Wearable Electrocardiogram Sensor. Polymers.

[73] Song J., Kim Y., Kang K., Lee S., Shin M., Son D. (2022). Stretchable and Self-Healable Graphene–Polymer Conductive Composite for Wearable EMG Sensor. Polymers.

[74] Choi Y., Park K., Choi H., Son D., Shin M. (2021). Self-Healing, Stretchable, Biocompatible, and Conductive Alginate Hydrogels through Dynamic Covalent Bonds for Implantable Electronics. Polymers.

[75] Koo J.H., Song J.-K., Kim D.-H., Son D. (2021). Soft Implantable Bioelectronics. ACS Materials Lett..

[76] Li G., Huang K., Deng J., Guo M., Cai M., Zhang Y., Guo C.F. (2022). Highly Conducting and Stretchable Double-Network Hydrogel for Soft Bioelectronics. Adv. Mater..

[77] Nishimura A., Suwabe R., Ogihara Y., Yoshida S., Abe H., Osawa S., Nakagawa A., Tominaga T., Nishizawa M. (2020). Totally Transparent Hydrogel-Based Subdural Electrode with Patterned Salt Bridge. Biomed. Microdevices..

[78] Terutsuki D., Yoroizuka H., Osawa S., Ogihara Y., Abe H., Nakagawa A., Iwasaki M., Nishizawa M. (2022). Totally Organic Hydrogel-Based Self-Closing Cuff Electrode for Vagus Nerve Stimulation. Adv. Healthcare Mater..

[79] Sun J.-Y., Zhao X., Illeperuma W.R.K., Chaudhuri O., Oh K.H., Mooney D.J., Vlassak J.J., Suo Z. (2012). Highly Stretchable and Tough Hydrogels. Nature.

[80] Hwang J.C., Kim M., Kim S., Seo H., An S., Jang E.H., Han S.Y., Kim M.J., Kim N.K., Cho S.-W. (2022). In Situ Diagnosis and Simultaneous Treatment of Cardiac Diseases Using a Single-Device Platform. Sci. Adv..

[81] Jin S., Kim Y., Son D., Shin M. (2022). Tissue Adhesive, Conductive, and Injectable Cellulose Hydrogel Ink for On-Skin Direct Writing of Electronics. Gels.

[82] Park J.W., Park K.H., Seo S. (2019). Natural polyelectrolyte complex-based pH-dependent delivery carriers using alginate and chitosan. J. Appl. Polym. Sci..

[83] Park K., Choi H., Kang K., Shin M., Son D. (2022). Soft Stretchable Conductive Carboxymethylcellulose Hydrogels for Wearable Sensors. Gels.

[84] Jin Y., Yang T., Ju S., Zhang H., Choi T.-Y., Neogi A. (2020). Thermally Tunable Dynamic and Static Elastic Properties of Hydrogel Due to Volumetric Phase Transition. Polymers.

[85] Yang N., Wong K.K.H., de Bruyn J.R., Hutter J.L. (2008). Frequency-Dependent Viscoelasticity Measurement by Atomic Force Microscopy. Meas. Sci. Technol..

[86] Choi Y., Kang K., Son D., Shin M. (2022). Molecular Rationale for the Design of Instantaneous, Strain-Tolerant Polymeric Adhesive in a Stretchable Underwater Human–Machine Interface. ACS Nano.

[87] Susanti E., Wulandari P., Herman (2018). Effect of Localized Surface Plasmon Resonance from Incorporated Gold Nanoparticles in PEDOT:PSS Hole Transport Layer for Hybrid Solar Cell Applications. J. Phys. Conf. Ser..

[88] Xu Z., Song J., Liu B., Lv S., Gao F., Luo X., Wang P. (2021). A Conducting Polymer PEDOT:PSS Hydrogel Based Wearable Sensor for Accurate Uric Acid Detection in Human Sweat. Sens. Actuators B.

[89] McGlynn E., Nabaei V., Ren E., Galeote-Checa G., Das R., Curia G., Heidari H. (2021). The Future of Neuroscience: Flexible and Wireless Implantable Neural Electronics. Adv. Sci..

